# Effects of Tabata Training During Physical Education Classes on Body Composition, Aerobic Capacity, and Anaerobic Performance of Under-, Normal- and Overweight Adolescents

**DOI:** 10.3390/ijerph17030876

**Published:** 2020-01-30

**Authors:** Jarosław Domaradzki, Ireneusz Cichy, Andrzej Rokita, Marek Popowczak

**Affiliations:** 1 Department of Biostructure, Faculty of Physical Education, University School of Physical Education in Wrocław, al. I.J. Paderewskiego 35, 51-612 Wrocław, Poland; jaroslaw.domaradzki@awf.wroc.pl; 2 Department of Team Sports Games, Faculty of Physical Education, University School of Physical Education, al. I.J. Paderewskiego 35, 51-612 Wrocław, Poland; ireneusz.cichy@awf.wroc.pl (I.C.); andrzej.rokita@awf.wroc.pl (A.R.)

**Keywords:** high-intensity interval training, body mass index, health-related fitness, students

## Abstract

Physical education classes often fail to include sufficient exercise intensity to induce changes in body tissue composition and physical fitness. Short-term high-intensity interval training protocols incorporated into physical education lessons are one possible solution to this problem. Existing studies have not examined how individuals differing in body mass index (e.g., normal-weight, underweight) respond to high-intensity interval training exercises. Therefore, this study aimed to evaluate the effects of a Tabata protocol on body composition measurements, aerobic capacity, and motor performance in underweight and overweight adolescents (the experimental groups) vs normal-weight adolescents (here regarded as the control group). The sample included 58 adolescents (28 boys, mean age = 16.2 years; 30 girls, mean age = 16.2 years) who completed the high-intensity interval training and the following set of measurements pre- and post- intervention: height, weight, body fat percentage and waist-to-hip ratio, physical efficiency index (based on the Harvard Step Test), agility (in 4 × 10 shuttle run test), and lower-limb muscle power in vertical jump. The results showed that high-intensity interval training was effective in reducing body weight, waist-to-hip ratio, and body fat percentage only in overweight individuals. Improvement in aerobic capacity was found only in underweight and overweight boys. Further research should focus on the development of customized exercise protocols and their adaptation to girls and assess the sustainability of the changes induced.

## 1. Introduction

Malnutrition in all its forms, including overweight, obesity, and underweight forms, is the primary cause of global health deterioration. Currently, over-nutrition and undernutrition (especially in third world countries) are widespread across the globe and affect individuals in every country and region of the world [1]. Both malnutrition problems (over- and underweight) are affecting healthcare costs. Scientific evidence suggests that the global trend of a steadily increasing percentage of both overweight and underweight individuals is increasingly affecting children and young people [2,3].

Overweight, obesity, and underweight forms in childhood and adolescence may bring about numerous consequences and lead to health problems in adulthood. Being underweight increases the risk of infectious diseases. In girls, it often causes menstrual cycle disorders and increases the risk of miscarriage, preterm birth, and faster involution of the reproductive system in adulthood [4,5,6]. Being overweight or obese in childhood often persists in adulthood [7] and leads to numerous diseases of affluence, such as type-2 diabetes, cardiovascular diseases, and metabolic diseases, and, consequently, leads to premature death [8,9,10,11].

Body weight is linearly related to body mass index (BMI). For this reason, BMI is an essential biological measure of a population’s biological condition and related social phenomena [12]. Due to its strong relationship with physiological markers, BMI is a predictor of diseases of affluence. The categorization of BMI allows for the identification of subsets of the population prone to different health complications.

Many researchers have focused on finding optimal methods to combat obesity and overweight. Results have shown that one of the most important and effective methods to address obesity and overweight is physical activity [13,14]. Actions taken to address the problem of malnutrition include the development of programs for the promotion of physical activity and their implementation in physical education (PE) in schools. PE classes are considered an ideal setting to promote healthy physical activity and prevent overweight and obesity [15], helping to ensure that physical activity becomes more frequent among all students. 

However, studies on the benefits of such programs are inconclusive. Different degrees of effectiveness have been shown depending on the type of exercise, the form of the classes, and the range of parameters analyzed. The most common effects are those related to the reduction of BMI and body fat and, sometimes, improvement in physical fitness in obese and overweight individuals [16]. However, no studies have examined underweight individuals, for whom a reduction in body weight or body fat would be undesirable. Physical fitness tests should be introduced into health monitoring systems—Ortega et al. [17], especially those testing the efficiency of the cardio-respiratory system, the base for physical efficiency, and muscular fitness and speed-agility, the base for motor performance [18].

It also remains unclear what type of program, type of exercise, duration, and volume constitute optimal training for inducing the desired changes in adolescents. The results of a few studies have indicated that high-intensity interval training (HIIT) may be appropriate. The training is based on a short intervention time (up to several minutes) of very intensive effort (from 75% HRmax). Such intensity in this training improves the maximum oxygen uptake in adolescents [19]. The effects of this protocol have been presented by numerous authors [19,20,21,22,23]; however, such analyses were carried out in groups of young people without consideration of the variation in their BMIs. Determination of the impact and effect size of HIIT in children and adolescents with different BMIs should be a basic objective of research based on the principle of individualization, including the division of young people into not only normal-weight, overweight, and obese but also underweight groups. Furthermore, the effectiveness of programs conducted in natural conditions (i.e., during PE lessons at school) also needs to be verified. In searching for novelty in the area of studies on HIIT programs in PE, we determined that there appear to be no studies in which participants were separated into underweight, normal-weight, and overweight groups. Therefore, this study aimed to evaluate the effects of a HIIT program based on the Tabata procedure on body composition, aerobic capacity, and motor performance in underweight, normal-weight, and overweight adolescents (divided by gender). The working hypothesis of the present study was that the HIIT intervention would affect body composition, aerobic capacity, and motor performance independently of BMI status. Different gender results are expected also.

## 2. Methods

### 2.1. Participants

Participants comprised boys and girls aged 16 years (see details below) from a Polish general secondary school. The students who attended the school were of the same sociocultural level and lived in the same geographical area (i.e., a big city with about 650,000 inhabitants). In addition, they were recruited on the basis of two inclusion criteria. The first criterion required being in the first year of general secondary school (10th grade), during which the HIIT program was introduced. The second criterion was that parental consent had to be obtained to participate in this research. Exclusion criterions were metabolic diseases or asthma. It was implicated of medical contraindications.

High-intensity interval training (HIIT) is generally a form of interval training alternating short periods of intense anaerobic exercise with less intense recovery periods, until the participant is too exhausted to continue. The students followed a 14-min HIIT exercise regimen based on the Tabata training method, presented in the form of a video during one of three PE lessons per week. Tabata training takes its name from Izumi Tabata, who examined changes in the aerobic and anaerobic systems after high intensive interval training (HIIT) utilizing his own protocol.

The Tabata protocol was followed for a period of one school semester (10 weeks). The remaining PE lessons were conducted in accordance with the PE curriculum adopted by the school for first-year students. The diets of the participants were not analyzed, but students were instructed to maintain their diet and normal level of activities of daily living and not to engage in any other organized physical activity outside of the PE classes [24]. 

Participants comprised 39 boys and 45 girls who attended the Tabata program. The analysis was based on data from 28 boys (mean age= 16.2 years, SD= 0.4) and 30 girls (mean age= 16.2, SD= 0.4) who completed the HIIT program as well as a set of measurements before and after the intervention and who were not excluded on the basis of the aforementioned inclusion criteria. Data were excluded from participants involved in organized physical exercise (i.e., a fitness gym) or additional recreational activities during the previous six months (three girls, four boys), those who had medical contraindications for motor activity and/or cardiovascular and respiratory diseases (three girls, one boys), those who discontinued participation in PE classes (as a result of school or group/class change; five girls, four boys), and those who failed to complete all tests (five girls, two boy). No participants withdrew from the program due to fatigue or lack of interest. The included participants were divided into three groups: underweight (BMI < 18.5 kg/m^2^), normal-weight (BMI = 18.5–24.99 kg/m^2^), and overweight (BMI ≥ 25 kg/m^2^). Underweight and overweight adolescents were regarded as experimental groups and were compared with normal-weight adolescents, who were regarded as the control group.

The study was approved by the Ethics Committee of the University of the Physical Education in Wroclaw (ECUPE No. 33/2018). The study was also conducted in accordance with the ethical principles for medical research involving human subjects contained in the Declaration of Helsinki by the World Medical Association. Additionally, the study met the “ethical standards in sport and exercise science research” [25]. All participants and their parents were asked to provide written informed consent prior to the study. 

### 2.2. Procedures

Morphological and motor measurements were performed before and after the 10-week intervention. The tests were conducted on one day, from 8:00 a.m. to 1:00 p.m., in sports halls in standard conditions for each group. Each participant was wearing a T-shirt, shorts, and shoes. Only anthropometric measurements were conducted without shoes. The measurements were performed in the following order: anthropometric measurements, vertical jump, 4 × 10 m shuttle run agility, and the Harvard Step Test. The protocol used for each measurement was based on recommendations for the assessment of health- and skill-related physical fitness [26]. 

### 2.3. Anthropometric Measurements

Morphological measurements included body height, with an accuracy of 0.1 cm, using anthropometers (GPM Anthropological Instruments, DKSH Ltd, Switzerland) and body weight and body fat percentage (BF%) using an InBody230 body composition analyzer (InBody Co. Ltd, Cerritos, CA, USA) with the bioelectrical impedance method. The tool has very high reliability. InBody230 was reliable in men and women as indicated by high intraclass correlation coefficients for BF% (≥0.98), FM (≥0.98), and FFM (≥0.99) and low standard error of measurement [27]. Before the measurement, participants were asked to excrete, refrain from drinking excessive amounts of water and not change typical breakfast patterns. BMI was calculated based on body height and weight. Furthermore, waist-to-hip ratio (WHR) was calculating.

### 2.4. Aerobic Capacity (Harvard Step Test)

Aerobic capacity was assessed using the Harvard Step Test. Physical efficiency index (PEI) was next calculated. Reliability of this test is acceptable at the intraclass correlations coefficient (ICC) = 0.63 [28]. The Harvard Step Test is very useful because it requires minimal equipment, no calibration, and is possible to be conducted indoor. From the school class point of view, it takes only 8 minutes (5 minutes of exercise, 3 minutes of monitored recovery) to complete, in addition, several people can be measured simultaneously. The participants stepped up and down at a constant pace on a stool with a height of 41.3 cm. The process began with the participants stepping onto and off of the step box at a pace of 30 cycles per minute with a metronome set at 120 beats per minute (bpm). The exercise was performed for a period of 300 s, less if the participants were compelled to stop owing to fatigue. The recovery pulse was recorded as the bpm during the 1 min after completing the test. Prior to each test, heart rate monitors (Polar H1, Polar Electro; Kempele, Finland) were fitted to each schoolchildren’s chest, level with xiphoid process and underneath exercise attire. Resting heart rate as well as changes in heart rate during exercise and 1.5 min after the test concluded (i.e., the recovery pulse) were measured. Heart rate monitors sampled participant responses at 5-s intervals and were transmitted to a smartwatch (Polar, Polar Electro; Kempele, Finland).

The PEI was calculated using the following formula [29]: PEI = (100 × L)/(5.5 × p), where L is the duration of the test (L = 300 s) and p is the number of heartbeats in the 1.5 min after the participant completed the test.

### 2.5. Motor Performance

The following tests were used to determine the motor component: the 4 × 10 m shuttle run test to evaluate agility and the vertical jump test to evaluate the power of the lower limbs. 

The 4 × 10 m shuttle run test (agility) was performed according to a previous description [30]. Two parallel lines were drawn on the floor 10 m apart. The participants ran as fast as possible from the starting line to the other line and returned to the starting line, crossing each line with both feet every time. The researcher was situated at the starting line and stopped the stopwatch when the participants crossed the line with one foot. The time taken to complete the test was recorded to the nearest tenth of a second. Participants wore sports shoes and performed the test twice with a 5-min rest in between, and the best time was selected.

The power of the lower limbs was assessed using the static vertical jump procedure in the vertical jump test, in which a static position with a knee flexion angle of 90° was maintained for 2 s before a jump attempt without any preparatory movement. All jumps were executed with the hands on the hips. Three jump attempts were performed, separated by 15-s intervals. The jumps were performed on a g-force tracker (Vert Jump; VERT, Fort Lauderdale, FL, USA) with the obtained flight time (t) being used to estimate the height of the rise of the body’s center of gravity (h) during the vertical jump (i.e., h = gt^2^/8, where g = 9.81 m/s^2^). The best attempt was used in our data analysis.

All measurements as measured in our study parameters met H-RF (health-related fitness) conception and constitute morphological, muscular fitness and endurance, cardio-vascular endurance, and motor abilities (speed and agility) health-related fitness components. The scientific rationale for the selection of all of these tests, as well as their reliability in young people, have previously been published [17,27,28].

### 2.6. Intervention

During one PE lesson per week, participants followed the HIIT exercise regimen based on the Tabata training protocol [31]. A PE lesson in the current intervention started with organizational activities in the group and motivating students to actively participate. Next, a standardized 10-min warm-up consisting of 5 min of slow jogging followed by 5 min of stretching (dynamic and static) of major muscle groups was performed. The main part of the lesson was the HIIT training based Tabata protocol, with a total time of 14 min. This training was divided into three sessions, each lasting 4 min. Each session based Tabata protocol (20 s work/10 s rest) consisted of eight cycles of two exercises. Each cycle started with a maximum intensity exercise lasting for 20 s, in which the participant was motivated to perform as many repetitions as possible of a given exercise involving large muscle groups of the entire body (np. narrow stance squat, butt kicker, toe touches, lunges, mountain climbers, jumping jacks, standing abs twist, squat to side), which was followed by a 10-s active rest in the form of a low-intensity exercise (i.e., skipping without rope, jumping without rope, walking). The cycles were repeated without any rests between them. The total duration of each session was 4 min. There was a 1-min passive rest between each session during which no exercises were performed. After the Tabata training, the final part of the training, including flexibility and relaxation exercises, was performed for several minutes. All the exercises were prepared by the authors (for the purpose of the experiment), recorded, and played during the PE lesson on a screen to ensure that the times of exercise and rest were implemented accurately.

To verify the exercise intensity during the Tabata training, the maximum heart rate (HR_max_) of the participants was determined according to the formula proposed by Tanaka, et al. [32] HR_max_ = 208 − 0.7 × “age” (age = 16 years in this study). A calculated HR_max_ of 197 bpm was used to calculate the high-intensity exercise range of 75%–80% (145–157 heartbeats/min). During the PE lesson with Tabata training, the effects of the program were monitored using HR measured by a Polar H1 heart rate monitor (Polar Electro, Kempele, Finland). The HR was displayed on the Polar H1 watch screen to motivate the students to maintain adequate exercise intensity. On average, the participants achieved an HR of 156.2 bpm (standard deviation, SD *=* 17.8; confidences intervals, 95% CI): 123.0–184.0) 

### 2.7. Statistics

The Shapiro–Wilk test was used to evaluate the normality of the distribution of data, and all variables showed a normal distribution. Descriptive data were presented as means and standard deviations. A two-way analysis of variance (ANOVA) was used to evaluate pre-training differences between groups (BMI status: underweight, normal-weight, overweight; gender: boys and girls). Next three-way analysis of variance (ANOVA) with repeated measures was performed (pre/post × BMI status × gender) to determine if there were any between-group changes as a result of the training. When significant differences were observed (significant *F*-ratio), detailed comparisons of post-hoc tests (Tukey’s HSD test) were used to determine pairwise differences. The significance level was set at α= 0.05. Cohen’s *d* values were calculated as a measure of effect size for mean differences with the following interpretation: above 0.8 were considered as large, between 0.79 and 0.5 (medium), between 0.49 and 0.2 (small) and lower than 0.2 (trivial) [33]. We used Statistica version 13.0 program (StatSoft Polska, Cracow, Poland) to analyze the study data.

## 3. Results

Descriptive statistics of the parameters for boys and girls from the different BMI groups are presented in Table 1.

Analysis of variance showed statistically significant interaction between factors (gender × BMI groups) was statistically significant (Λ = 0.3623, *F* = 2.53, df effect= 22, p = 0.0013, η_p_^2^ = 0.3981). Statistically significant were also main effects—variation between gender (Λ = 0.2131, *F* = 14.10, df effect= 11, *p* < 0.001, η_p_^2^ = 0.7869) and BMI groups (Λ = 0.0984, *F* = 8.36, df effect= 22, *p* < 0.001, η_p_^2^ = 0.6864).

The two-way ANOVA found significant differences before training according to BMI status and gender, but the two factors did not interact with each other. BMI groups differed in body weight (*F* = 89.52, *p* < 0.001), BMI (*F* = 120.30, *p* < 0.001), WHR (*F* = 59.18, *p* < 0.001), and BF% (*F* = 47.10, *p* < 0.001). Furthermore, BMI groups differed in vertical jump results (*F* = 4.055, *p* = 0.023). No other significant differences between the groups were observed for other variables. 

Detailed comparisons showed the gender dimorphism of basic morphological features, as boys and girls differed in body height (*F* = 54.12, *p* < 0.001) and body weight (*F* = 17.50, *p* < 0.001). The gender differences in body fat were similar in terms of WHR (*F* = 59.19, *p* < 0.001) and BF% (*F* = 47.096, *p* < 0.001). Gender differences were also found in agility (*F* = 32.83, *p* < 0.001) and the vertical jump (*F* = 32.80, *p* < 0.001). 

The analysis of differences according to BMI status indicated that boys and girls from overweight groups were characterized by higher levels of body weight, BMI, WHR, and BF% compared with both slimmer groups, while underweight groups of both genders achieved lower body weight and BMI compared with normal-weight groups. However, in the vertical jump test, the significant differences concerned only underweight and overweight boys (Table 2).

Using ANOVA with repeated measures, the comparison of the results before and after the intervention revealed highly significant effects of HIIT on body weight (*F* = 22.24, *p* < 0.001), BMI (*F* = 13.89, *p* < 0.001), WHR (*F* = 32.92, *p* < 0.001), and BF% (*F* = 14.56, *p* < 0.001). The intervention also demonstrated significant changes in aerobic capacity (PEI; *F* = 17.523, *p* < 0.001) and agility (*F* = 6.95, *p* = 0.010). The results of detailed comparisons using Tukey’s HSD test before and after the intervention are presented in Table 1. 

HIIT induced changes in anthropometric parameters (body weight, BMI, WHR, BF%), which almost exclusively concerned overweight boys and girls (Table 1). The improvement of aerobic capacity was also observed in underweight and normal-weight boys. 

Comparisons between the groups following the intervention revealed the persisting direction of differences observed before training. Overweight boys and girls continued to differ from other groups; however, the differences were smaller than prior to the intervention. 

## 4. Discussion

The HIIT program based on the Tabata protocol produced partial effects that can be considered from two standpoints. First, the changes concerned mainly overweight groups, both boys and girls. Second, the changes involved morphological rather than functional features. The effects included significant reductions in body weight, BMI, WHR, and body fat in overweight adolescents. However, additional improvements in aerobic performance were observed in both underweight and overweight boys. 

Our results are partially consistent with the findings of other researchers [20,22,23] who also documented positive effects of HIIT among overweight and obese people. In our study, we did not find any changes in body composition in underweight young people, which can be considered a positive pattern. Therefore, the proposed HIIT regimen seems to be safe for adolescents. In this respect, our results are consistent with previous studies [34,35] that found no decrease in BMI or BF% in underweight adolescents [36,37].

All analyzed morphological parameters constitute a morphological health-related fitness component. In this sense, the HIIT regimen can be considered to support the health of young people. In this study, its efficacy and usefulness in natural conditions (i.e., PE classes) were verified. The implementation of such training programs and scientific verification of their effects in the school environment are not easy, with difficulties including the organization of school work, organization of lessons, attitudes of young people, diversity of the sports facilities, and curriculum requirements, which may explain the small number of studies that have addressed this topic. Furthermore, not all studies have documented positive effects of programs integrated into school PE curricula [35].

There is still scarce information about the impact of various physical activity programs, especially regarding the effects of interval training with high or medium intensity on aerobic and anaerobic performance, and few studies have examined the effects on individuals with different BMIs. Such studies currently constitute one of the most important fields of research because of the growing percentage of overweight and obese people in Polish, European, and global populations, but they also concern underweight youth [38,39,40,41,42]. 

In general, correlations between BMI and the level of physical fitness of young people with different BMI statuses have been documented [43]. However, studies on the effects of HIIT programs on aerobic performance are inconclusive. Some researchers have shown the effects of such protocols on improvements in maximal oxygen uptake in both normal-weight and overweight young people [20,21]. However, the results of our research do not fully confirm the benefits of the 10-week interval training program, as we found no significant changes in aerobic capacity in the groups of girls. A similar pattern was found in the group of normal-weight boys, although their body weight and BMI decreased significantly. However, some researchers have demonstrated the effects of interval training programs, such as weight loss and reduced BMI [44,45]. Our results also differ from those of a previous study [46] that observed improved cardiopulmonary efficiency in overweight and obese individuals. It is difficult to evaluate these discrepancies unequivocally. The lack of convergence of the results may be due to differences between our Tabata protocol and other interval exercises performed in HIIT. 

We also found no improvements in anaerobic performance based on the results of the vertical jump test (power of lower limbs) and shuttle run test (agility). In our opinion, this can be explained by the fact that the interval training was focused on aerobic changes. However, our results differ from those published in previous work [14,20], which showed a positive impact of interval training on anaerobic markers (vertical jump and 10-m sprint). Other studies also reported improvements in lower limb power during countermovement jumps and squat jumps, which were not related to weight reduction [47]. The increase in power can be explained by the increased activity of the anaerobic glycolysis enzymes in skeletal muscles following interval training [48].

Our research has certain limitations. First, the number of participants was small. Second, there was no dietary control for the participants. It remains unclear whether the participants maintained their dietary preferences and what the energy supply was during the implementation of the 10-week HIIT (Tabata) training program. Third—there was a lack of a control group. One of the inclusion criteria was performing no other structured exercise during participants’ leisure time. However, it cannot be guaranteed that participants did not undertake any additional physical activity during the study period. In the future, participants should use accelerometers to monitor their daily physical activity. Moreover, research on the effectiveness of interval protocols included in PE classes should also consider nutritional aspects. 

## 5. Conclusions

The division of participants into underweight, normal-weight, and overweight (obese) groups allows researchers to capture the actual directions of changes and effects of the programs used. Our study documented a significant reduction in body weight and BMI as well as redistribution and reduction of body fat, but only in groups of overweight boys and girls. Furthermore, underweight and overweight boys improved their aerobic performance. If the improvement in overweight boys can be attributed to weight loss, this improvement in underweight boys (with no weight loss) can be viewed as a net effect of the Tabata program. Moreover, training had no effect on anaerobic performance. 

In conclusion, the Tabata program represents a useful tool for combating the problem of being overweight while avoiding adverse effects (in terms of weight and body fat reduction) among underweight and normal-weight individuals, for whom weight reduction is not desirable. However, further research should be oriented toward the definition of rules for HIIT protocols addressed to genders and groups with different BMI to induce a general improvement in aerobic performance. 

## Figures and Tables

**Table 1:** Descriptive statistics of the anthropometric, physical efficiency index (PEI), and motor performance measurements and effect sizes and *p*-values (from Tukey’s test) for differences before and after the 10-week Tabata training (only statistically significant results) in adolescent boys and girls by BMI status.

Variable	BMI Status	Boys						Girls					
		Pre		Post		Cohen’s *d*	*p*	Pre		Post		Cohen’s *d*	*p*
		Mean	SD	Mean	SD			Mean	SD	Mean	SD		
BH [cm]	U	176.62	6.41	176,62	6,41	0,00	ns	164.95	4.71	164.95	4.71	0,00	ns
	N	177.24	5.96	177,68	6,90	0,07	ns	163.48	6.82	163.48	6.82	0,07	ns
	O	178.70	6.38	178,70	6,38	0,00	ns	166.41	7.62	166.41	7.62	0,00	ns
BW [kg]	U	53.90	2.58	54.49	3.11	0,21	ns	48.44	3.20	48.24	2.78	0,07	ns
	N	67.62	4.97	66.63	5.36	0,19	ns	58.48	5.46	59.03	5.66	0,10	ns
	O	89.12	9.06	84.80	10.50	0,44	<0.001	79.04	13.63	76.67	14.90	0,17	0.020
BMI (kg/m^2^)	U	17.30	1.00	17.49	0.96	0,19	ns	17.79	0.71	17.73	0.53	0,10	ns
	N	21.46	0.60	21.14	0.73	0,48	ns	21.85	0.59	22.05	0.72	0,31	ns
	O	27.51	2.99	26.67	2.91	0,28	0.012	28.57	3.91	27.62	4.26	0,23	0.017
WHR	U	0.79	0.01	0.78	0.02	0,67	ns	0.83	0.03	0.81	0.03	0,67	ns
	N	0.81	0.03	0.78	0.04	0,86	<0.001	0.85	0.04	0.86	0.05	0,22	ns
	O	0.93	0.04	0.89	0.05	0,89	0.002	0.95	0.06	0.91	0.06	0,67	0.001
BF [%]	U	10.50	3.00	9.28	2.65	0,43	ns	23.41	2.54	22.38	2.52	0,41	ns
	N	15.10	4.18	13.15	5.43	0,41	ns	27.92	5.79	28.47	5.72	0,10	ns
	O	27.58	6.02	24.82	5.27	0,49	0.039	36.76	5.36	34.79	5.04	0,38	<0.001
PEI [*n*]	U	41.63	4.54	45.72	2.93	1,10	0.016	42.00	5.31	46.42	5.11	0,85	ns
	N	42.36	2.59	44.23	1.86	0,84	ns	44.52	4.82	47.09	5.97	0,48	ns
	O	41.45	4.16	46.70	3.93	1,30	0.024	42.39	3.78	44.73	5.03	0,53	ns
Ag [s]	U	10.04	0.59	10.14	0.87	0,14	ns	11.64	0.77	11.87	0.70	0,31	ns
	N	9.75	0.35	9.85	0.68	0,19	ns	11.44	0.92	11.51	0.74	0,08	ns
	O	10.99	0.75	11.29	0.87	0,37	ns	11.12	0.83	11.35	0.78	0,29	ns
VJ [cm]	U	55.67	9.30	52.92	6.66	0,34	ns	41.75	6.86	41.74	5.35	0,00	ns
	N	58.40	7.32	59.97	8.06	0,20	ns	43.85	7.49	43.25	8.15	0,08	ns
	O	47.22	8.50	47.74	9.63	0,06	ns	41.67	4.09	42.21	5.96	0,11	ns

Note: SD: standard deviation, Cohen’s *d*: effect size pre and post intervention, ns: not significant; U: underweight, N: normal-weight, O: overweight; BH: body height, BW: body weight, BMI: body mass index, WHR: waist-to-hip ratio, BF%: body fat percentage, PEI: aerobic capacity, Ag: agility, VJ: vertical jump.

**Table 2:** P-values from Tukey’s test for differences between adolescents in different BMI status groups in pre- and post-tests (only statistically significant results).

Variable	Test	Boys			Girls		
		U–N	U–O	N–O	U–N	U–O	N–O
BW	pre	0.002	<0.001	<0.001	0.042	<0.001	<0.001
	post	ns	<0.001	<0.001	ns	<0.001	<0.001
BMI	pre	0.001	<0.001	<0.001	0.001	<0.001	<0.001
	post	0.013	<0.001	<0.001	0.001	<0.001	<0.001
WHR	pre	ns	<0.001	<0.001	ns	<0.001	<0.001
	post	ns	<0.001	ns	ns	<0.001	ns
BF	pre	ns	<0.001	<0.001	ns	<0.001	<0.001
	post	ns	<0.001	<0.001	ns	<0.001	ns
PEI	pre	ns	ns	ns	ns	ns	ns
	post	ns	ns	ns	ns	ns	ns
Ag	pre	ns	ns	ns	ns	ns	ns
	post	ns	ns	0.005	ns	ns	ns
VJ	pre	ns	ns	0.022	ns	ns	ns
	post	ns	ns	ns	ns	ns	ns

Note: U: underweight; N: normal-weight; O: overweight; BH: body height; BW: body weight; BMI: body mass index; WHR: waist-to-hip ratio; BF%: body fat percentage; PEI: aerobic capacity; Ag: agility; VJ: vertical jump; ns: not significant.
